# Speech token detection and discrimination in individual infants using functional near-infrared spectroscopy

**DOI:** 10.1038/s41598-021-03595-z

**Published:** 2021-12-14

**Authors:** Darren Mao, Julia Wunderlich, Borislav Savkovic, Emily Jeffreys, Namita Nicholls, Onn Wah Lee, Michael Eager, Colette M. McKay

**Affiliations:** 1 The Bionics Institute, 384-388 Albert St, East Melbourne, VIC 3002 Australia; 2 Department of Medical Bionics, University of Melbourne, Parkville, VIC 3010 Australia; 3 Faculty of Health Sciences, Centre for Rehabilitation and Special Need Studies, Universiti Kebangsaan Malaysia, 53200 Kuala Lumpur, Malaysia

**Keywords:** Auditory system, Imaging and sensing

## Abstract

Speech detection and discrimination ability are important measures of hearing ability that may inform crucial audiological intervention decisions for individuals with a hearing impairment. However, behavioral assessment of speech discrimination can be difficult and inaccurate in infants, prompting the need for an objective measure of speech detection and discrimination ability. In this study, the authors used functional near-infrared spectroscopy (fNIRS) as the objective measure. Twenty-three infants, 2 to 10 months of age participated, all of whom had passed newborn hearing screening or diagnostic audiology testing. They were presented with speech tokens at a comfortable listening level in a natural sleep state using a habituation/dishabituation paradigm. The authors hypothesized that fNIRS responses to speech token detection as well as speech token contrast discrimination could be measured in individual infants. The authors found significant fNIRS responses to speech detection in 87% of tested infants (false positive rate 0%), as well as to speech discrimination in 35% of tested infants (false positive rate 9%). The results show initial promise for the use of fNIRS as an objective clinical tool for measuring infant speech detection and discrimination ability; the authors highlight the further optimizations of test procedures and analysis techniques that would be required to improve accuracy and reliability to levels needed for clinical decision-making.

## Introduction

Speech discrimination assessment is a key component of the audiometric test battery. Speech discrimination ability informs the diagnostic process and management decisions such as device choice and optimization of programming parameters. Because of its functional relevance, speech discrimination is also a helpful tool in patient counselling, and clinicians may consider assessment of speech discrimination of equal or higher importance than assessment of audiometric hearing thresholds. For example, speech identification performance, rather than hearing thresholds, is a key criterion for cochlear implantation in adults^1–3^. However, currently there is no reliable and clinically-feasible behavioral or objective method of determining speech sound discrimination in infants^4^. In the present study, we investigated whether functional near-infrared spectroscopy (fNIRS) is a suitable tool for assessing speech sound detection and discrimination in individual infants.

Early intervention for hearing-impaired infants is important for maximizing the development of oral speech and language skills^5,6^. The introduction of universal newborn hearing screening has greatly reduced the average age of diagnosis. However, in the absence of reliable and accurate behavioral measures of speech discrimination, the optimal choice of hearing instrument and its programming can take months or longer to be established in each individual. For example, the decision to proceed to cochlear implantation is often delayed until reliable behavioral language development information can be obtained with a hearing aid, and the hearing aid program itself can take some time for its parameters to be verified and optimized. Furthermore, it is imperative for a child to not only be able to detect speech sounds with their hearing instrument, but also discriminate between them. Therefore, a clinically viable objective speech detection and discrimination test would be an invaluable addition to the pediatric audiological test battery to fast-track device selection and programming and to facilitate ongoing monitoring of language development.

This study evaluated a method of objectively assessing speech detection and discrimination in sleeping infants using fNIRS. fNIRS is a neuroimaging technique that has been used in the research setting for applications in infants, including numerous studies relevant to language development^7,8^. It is a portable, cheap, and clinically viable alternative to functional magnetic resonance imaging. In addition, functional magnetic resonance imaging is highly prone to any motion artefact (incompatible with awake infants) and is very loud. fNIRS measures cortical activity in topographically distinct regions of interest, and thus is a suitable tool to investigate higher-order cortical processing such as that associated with speech discrimination or language understanding^9–11^.

Two other features of fNIRS make it an attractive potential tool for speech discrimination assessment in infants compared to techniques that use evoked potentials. First, the use of light in an imaging technique allows testing without interference from electrical artifacts, a particularly useful feature when assessing cochlear implant users^12–14^. Secondly, in infants with auditory neuropathy, evoked potentials are either impossible to measure or hard to interpret reliably, depending on the degree and site of pathology, limiting their usefulness in diagnosis and rehabilitation^15,16^. fNIRS can overcome this difficulty with auditory neuropathy, as it does not rely on neural synchrony, as does evoked potential measurements.

For over twenty years, researchers have sought an objective measure of speech sound discrimination, but such a measure suitable for routine clinical use has proven elusive. For example, the mismatch negativity (MMN) cannot be reliably measured in individuals^17–19^. The acoustic change complex (ACC) shows promise but can only detect change in two steady-state sounds (e.g., vowels and fricatives), so it has low ecological validity for speech understanding^20–22^. Both these cortical EEG measures are far more robust when the infant is awake, which presents significant practical problems for testing very young infants that tend to have naps throughout the day. The mismatch response (MMR) can be measured in sleeping babies but may not be reliable enough for clinical use^23^. fNIRS responses are larger and more widespread over various brain regions in sleeping infants^24^; combined with lower likelihood of motion artefacts during sleep, fNIRS is a very suitable imaging technique in young infants.

Next, a sound presentation strategy is needed for an objective speech discrimination test. A behavioral approach that has been used extensively in research settings to assess discrimination of sounds in infants uses a habituation-dishabituation paradigm, which measures the looking time of infants to standard and novel stimuli^25–27^. This paradigm relies on the phenomenon whereby the brain’s response habituates (i.e., reduces in amplitude) to repeated exposure of the same stimulus and then recovers to a strong response when a novel stimulus is presented. Nakano et al.^9^ used this habituation/dishabituation approach to measure fNIRS responses objectively in a large group of sleeping 3-month-old babies. They measured hemodynamic responses over temporal and prefrontal regions that were consistent with both detection and discrimination of speech sounds on a group level. Both regions of interest showed habituation to the repeated standard stimulus, i.e., a decay of response amplitude over time, and when a novel stimulus was introduced, these regions were strongly reactivated, indicating discrimination of novel from the standard speech token.

It is unclear, however, whether Nakano et al.’s^9^ test protocol would be suitable for obtaining reliable results in individual infants, since an extended procedure with significantly more blocks of novel and standard stimuli would be needed for statistical significance testing, but in return perhaps reducing the novelty of the novel stimulus. Over such repetitions, long-term adaptation may also occur, causing all responses to reduce in size over time, making it harder to extract responses from baseline noise and non-task-related activity.

In this study, we investigated whether extended and modified versions of the test protocol of Nakano et al.^9^ has potential for testing speech sound detection and discrimination in individual infants. Three different phonemic contrasts (pairs of consonant–vowel syllables) were tested and each individual’s fNIRS data were analyzed by extracting fNIRS hemodynamic responses to the speech sounds using a fully automatic signal processing pipeline. Discrimination of the speech sounds was assessed statistically on both group and individual data. We hypothesized that fNIRS responses could reveal when individual infants detect and discriminate speech sounds.

## Materials and methods

### Participants

Twenty-three healthy full-term infants (16 male; 7 female) who had passed newborn hearing screening or diagnostic audiology assessment, ranging in age from 2 to 10 months (average age 5.3 months; SD 2.7 months), participated in the present study. An additional three infants were recruited but excluded from the analysis due to having bilateral middle ear effusion (*n* = 1) or not sleeping within the allocated time (*n* = 2). While acknowledging that newborn hearing screening does not establish that a baby has normal hearing (it excludes significant hearing impairment) we considered these babies to be normally hearing.

Tympanometry (probe tone 226 Hz or 1000 Hz if under 6 months) was performed on the test day, and infants were required to have peaked tympanograms in at least one ear for inclusion. Tympanometry was not possible in three cases: participants 6, 7, and 8 were under 6 months of age at the time of testing and the necessary 1000 Hz probe tone tympanometer was not available at the time of testing. In these cases, inclusion in the study was based on normal otoscopic examination and no history of ear disease. English was the primary language for all infant participants’ parents.

This experiment was approved by the human ethics committee at the Royal Victorian Eye and Ear Hospital in Melbourne, Australia (project number 16/1261H). Data were collected at the Bionics Institute of Australia in accordance with human ethics committee guidelines. Informed consent was obtained from the parents of the babies before the start of the session.

### Stimuli

Speech sound tokens (consonant–vowel syllables) were produced by a native English (Australian) female speaker, recorded (44.1 kHz, 16 bit), trimmed to 500-ms duration, and equalized in RMS amplitude*.* Sound stimuli were presented to the participants bilaterally using ER3 insert-phones at 65 dB SPL.

Six speech tokens were paired into three speech sound contrast pairs varying in the number and type of discrimination cues and which we hypothesized would differ in discrimination difficulty: Baa/Tea, Boo/Bee and She/See. Baa/Tea differ in vowel identity, consonant voicing, and place of articulation, making multiple cues for discrimination. Boo/Bee differ only in vowel identity (a steady-state, broad spectral cue), and She/See differ only in consonant place of articulation (requiring finer temporal and spectral discrimination). For normal-hearing infants, as in this study, these contrasts are theoretically all discriminable, and we used an *assumption* of discrimination ability to quantify the accuracy of the fNIRS measurements.

For fNIRS measurements, we created five second stimulus block that consisted of a concatenation of ten identical 500 ms speech tokens. The first speech token of each pair listed above was used as the habituating stimulus and the second of each pair was the novel stimulus.

### Data acquisition

Participants were tested with the NIRScout continuous-wave fNIRS device manufactured by NIRx (NIRx Medical Technologies, LCC, USA). Near-infrared light was presented at two wavelengths (760 nm and 850 nm) and detected at 7.8125 Hz resolution.

**Figure 1:**
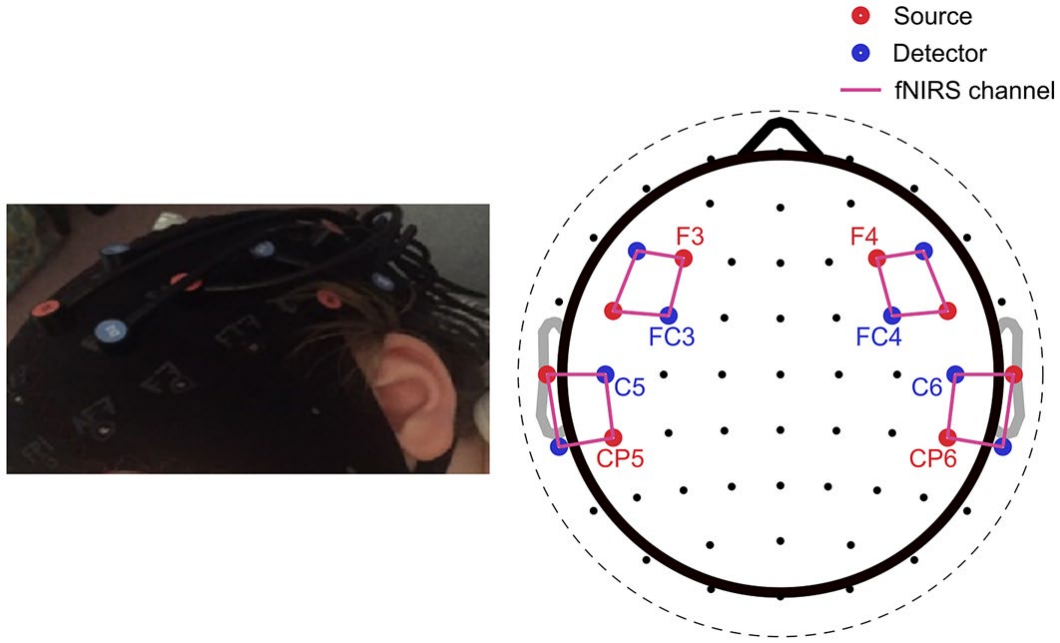
Illustration of experimental set-up on the infant. Left: an image of the cap on a participant’s head. Right: The 8 × 8 source-detector montage that was placed over the temporal and prefrontal regions of the infant participants, resulting in four regions of interest. Sources are colored red, detectors are blue, and channels are indicated by purple lines.

**Figure 2:**
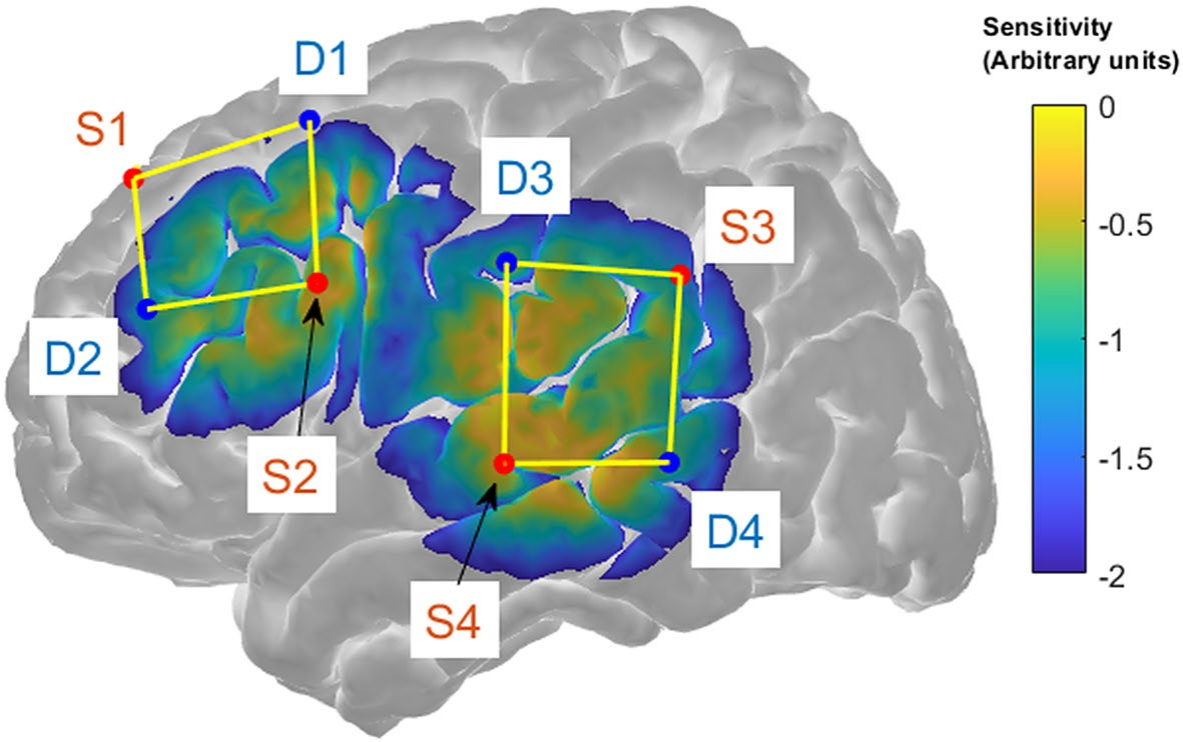
Sensitivity profile and sensor channel locations for the left temporal and prefrontal regions. The sensitivity of each probe to detecting brain hemodynamics is represented on a logarithmic color scale ranging from − 2 to 0 (arbitrary units; higher number indicates higher sensitivity). Source and detector positions are indicated by the red and blue circles and corresponding labels respectively, and yellow lines show channels. This figure was generated with AtlasViewer^29^.

Figure 1 shows the eight-by-eight source-detector montage, which was placed over the two regions of interest in temporal and prefrontal regions. A channel distance of 2–3 cm (found to be optimal for measuring infant hearing responses^28^) was used, and infant optodes (NIRx flat optodes) were used for babies under 12 months as per the NIRx specifications. The selected regions of interest were adapted from previous work by Nakano et al.^9^, who found different degrees of habituation in the temporal and prefrontal regions. Figure 2 shows the layout of the sources, detectors and channels overlaid on a brain image, along with the sensitivity profile for the left hemisphere^29^.

The head circumference of the baby was measured prior to testing, and an appropriately sized cap (EasyCap GmbH, Herrsching, Germany) was chosen accordingly. The EasyCap was correctly positioned using the nasion, inion, vertex, and tragus as landmarks, so that the optodes were located over 10/10 EEG electrode positions^30^ F3/4, F5/6, FC3/4, FC5/6 (prefrontal regions of interest on each hemisphere) and C5/6, T7/8, CP5/6, TP7/8 (temporal regions of interest on each hemisphere).

Testing was performed in a dimly lit, sound-attenuated booth. The parent sat in an armchair cradling their baby and were given time to feed and settle their baby before the experiment started. Measurements began once the baby was asleep.

The stimulus presentation procedure in each run followed that of Nakano et al.^9^ with the following adaptations to increase efficiency for individual assessments. The inter-block silent interval was reduced from 15 to 9 s, based on the fact that the average response shown in Nakano et al.^9^ returned to baseline before 9 s. Ten instead of fifteen blocks of the habituation stimulus were presented before the novel stimulus, based on our observation that average response in the last five of the fifteen habituation blocks (Nakano et al.’s “Hab 3”) did not differ significantly in size from that in the second group of five (Nakano et al.’s “Hab 2”).

**Figure 3:**
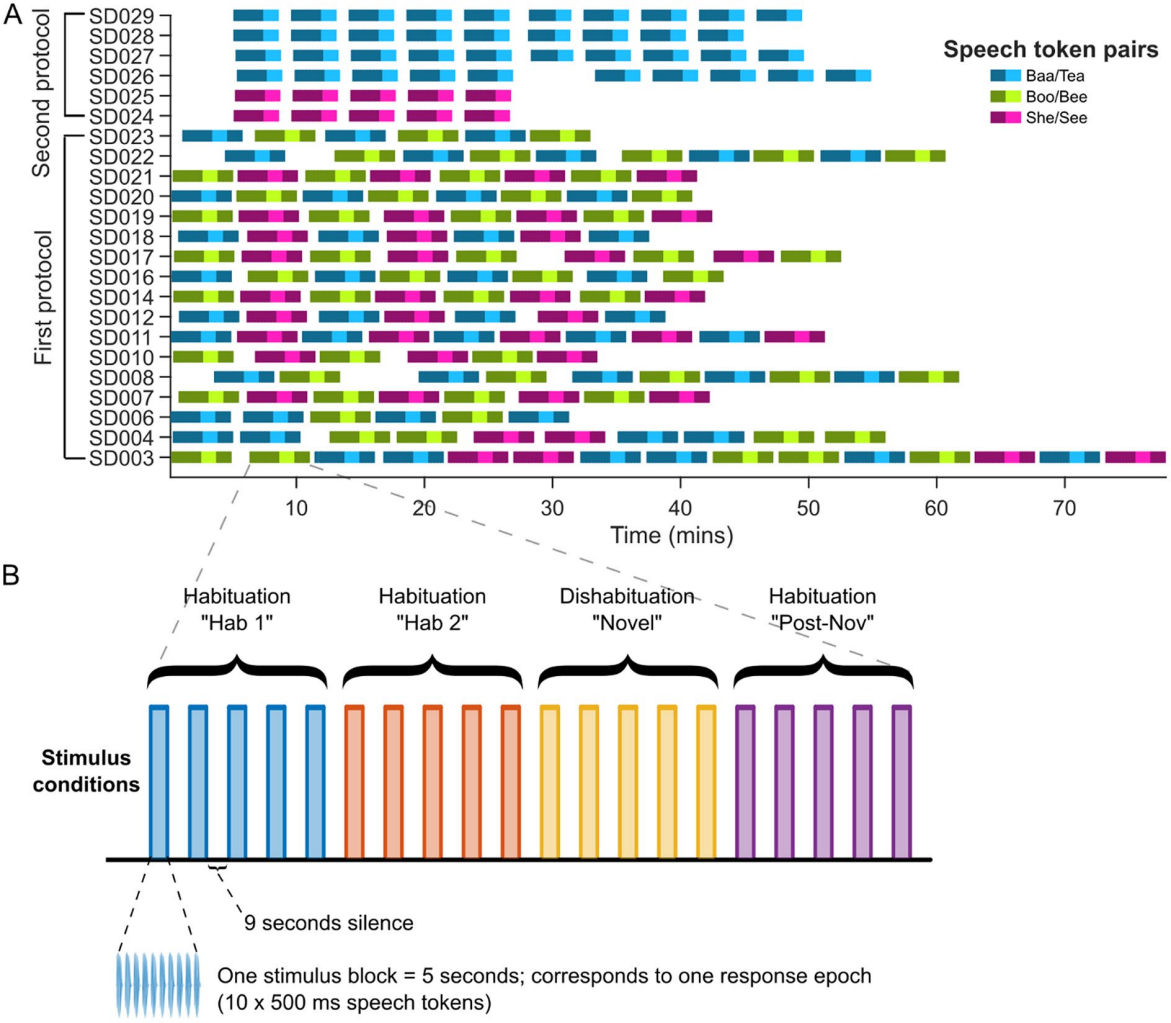
Representation of the sound presentation protocol used in this study. (**A**) Details of the test run sequence and timing for each infant in the study. Each two-color segment represents a test run of a specific speech contrast pair. Note that the final 6 infants received only one contrast pair, and the test run was modified to replace the “Post-Nov” condition with 1 min of silence. (**B**) Representation of the stimulus protocol for a single experimental test run. One run comprised four sections labeled as “Hab 1”, “Hab 2”, “Novel” and “Post-Nov”, each containing 5 stimulus blocks. The habituation speech sound was presented in the blocks labeled “Hab 1”, “Hab 2” and “Post-Nov”, while the novel speech sound was presented in the blocks labeled “Novel”.

The experiment consisted of multiple runs per participant (Fig. 3, panel A). For the first seventeen participants, an experimental run comprised four stimulus conditions (labeled Hab 1, Hab 2, Novel, and Post-Nov), each comprising five identical stimulation blocks followed by nine seconds of silence (Fig. 3, Panel B). The same habituation speech sound was presented in “Hab 1”, “Hab 2” and “Post-Nov” conditions, while the novel sound was presented in the “Novel” condition (Fig. 3; Panel A shows one band per run with Novel shown in a lighter color; Panel B shows the full breakdown of the run). Thus, one experimental run comprised five blocks of each of “Hab 1”, “Hab 2”, “Novel” and “Post-Nov”, and each full run took approximately five minutes to complete. Runs were repeated until the infant awoke, so the total recording time and total number of runs varied across infants, with the first 17 participants labeled as “first protocol”.

For the final six infants, labeled “second protocol” in Fig. 3 Panel A, the “Post-Nov” condition was replaced by 1 min of silence before the subsequent run started, with the aims of allowing the infant’s brain to return to rest state between runs and limiting long-term adaptation; since the post-novel condition was not used in the analysis of discrimination, its removal did not prevent us from comparing discrimination responses for the two presentation protocols. Note that a fixed inter-block interval was used (as in Nakano et al.) rather than the commonly used randomly varying interval, with the aim of enhancing habituation before the onset of the novel stimulus. Over all 23 participants, fNIRS recording times averaged 38.9 min (standard deviation 10.3 min). Baa/Tea was tested in 15 babies, Boo/Bee in 14, and She/See in 13.

Since these babies did not have any hearing disorder, it was expected that they would all be able to detect and discriminate the sounds. Therefore, we hypothesized that hemodynamic response to a speech syllable would be significantly greater than baseline (to determine significant detection) while the response to the “Novel” syllable would be larger than that to the habituated (“Hab 2”) syllable (to determine significant discrimination).

### Data pre-processing and analysis

**Figure 4:**
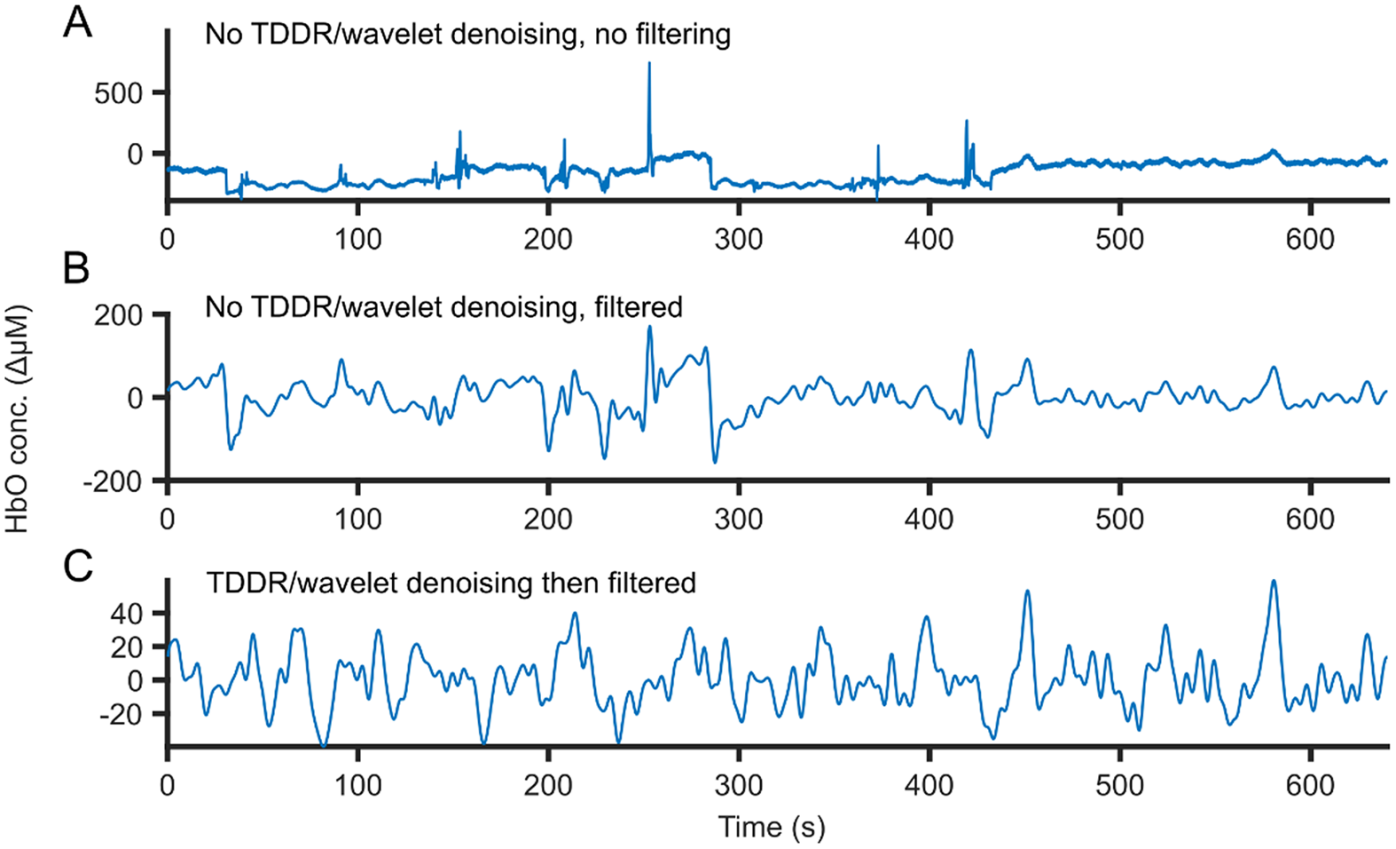
An example of TDDR and wavelet denoiser’s effect on the data. (**A**) The raw data processed with only optic density and modified beer-lambert law conversion, demonstrating spike-like artefacts from movements. (**B**) The data processed with only optical density, modified beer-lambert law and a bandpass filter (0.01 to 0.25 Hz), with the spikes still present and smoothed out from the filtering. (**C**) Data cleaned first by TDDR and wavelet denoising before standard processing as in (**A**), with visible reduction in artefacts.

fNIRS data were pre-processed using the NIRS Brain-AnalyzIR toolbox^31^ and a custom-written script. The pre-processing pipeline was fully automated, and thus required no hand-curation of data. Raw readings from the NIRScout device were converted to optical densities. Then, motion artefact correction was performed by first applying the temporal derivative distribution repair (TDDR) algorithm^32^ and then the wavelet denoiser^33^. A ‘sym8’ wavelet basis function was used with the outlier defined as values above 4 standard deviations. An example of the artefact correction is shown in Fig. 4. The modified Beer-Lambert Law^34^ was then applied to convert optical densities to concentrations of oxy- (HbO) and deoxy-hemoglobin (Hbr), creating HbO and Hbr time series. The correlation-based signal improvement (CBSI) method^35^ was applied to limit the systemic component of the fNIRS response; a consequence was that the HbO and Hbr data were now perfectly correlated, and thus only the HbO data were used for all analyses. Data were then band-pass filtered between 0.01 and 0.25 Hz (8th order Butterworth filters) to remove remaining physiological noise while preserving task-related responses^36^. Data were epoched from three seconds before stimulus onset to eleven seconds after stimulus onset, resulting in epochs 14 s in length. Data epochs were then baseline corrected and detrended by subtracting a linear fit to the first and last 3 s of the epoch.

The following automated data rejection methods were applied. On a per-channel basis, channels with a coefficient of variation (standard deviation divided by the mean) over the entire duration of the experiment of greater than 0.7 were rejected. Epochs that had a scalp coupling index of less than 0.75 (calculated as the correlation between the two optical wavelengths’ raw data in a frequency band between 1.5 and 3 Hz, containing the infant sleeping heart rate of approximately 120 beats per minute) were rejected. On a time-varying basis, a sliding window of 30 s in length was used to analyze the coefficient of variation over each data channel individually. Any segments that had a coefficient of variation greater than 1 were marked, and epochs that overlapped with these segments were rejected. Following data processing, the data was stored in a structure that contained individual epochs for each participant, recording channel, condition (Hab 1, Hab 2, Novel, Post-Nov) and repetition number. Using the above automatic rejection methods, on average 7.6% (range 3–15%) of epochs were rejected.

**Figure 5:**
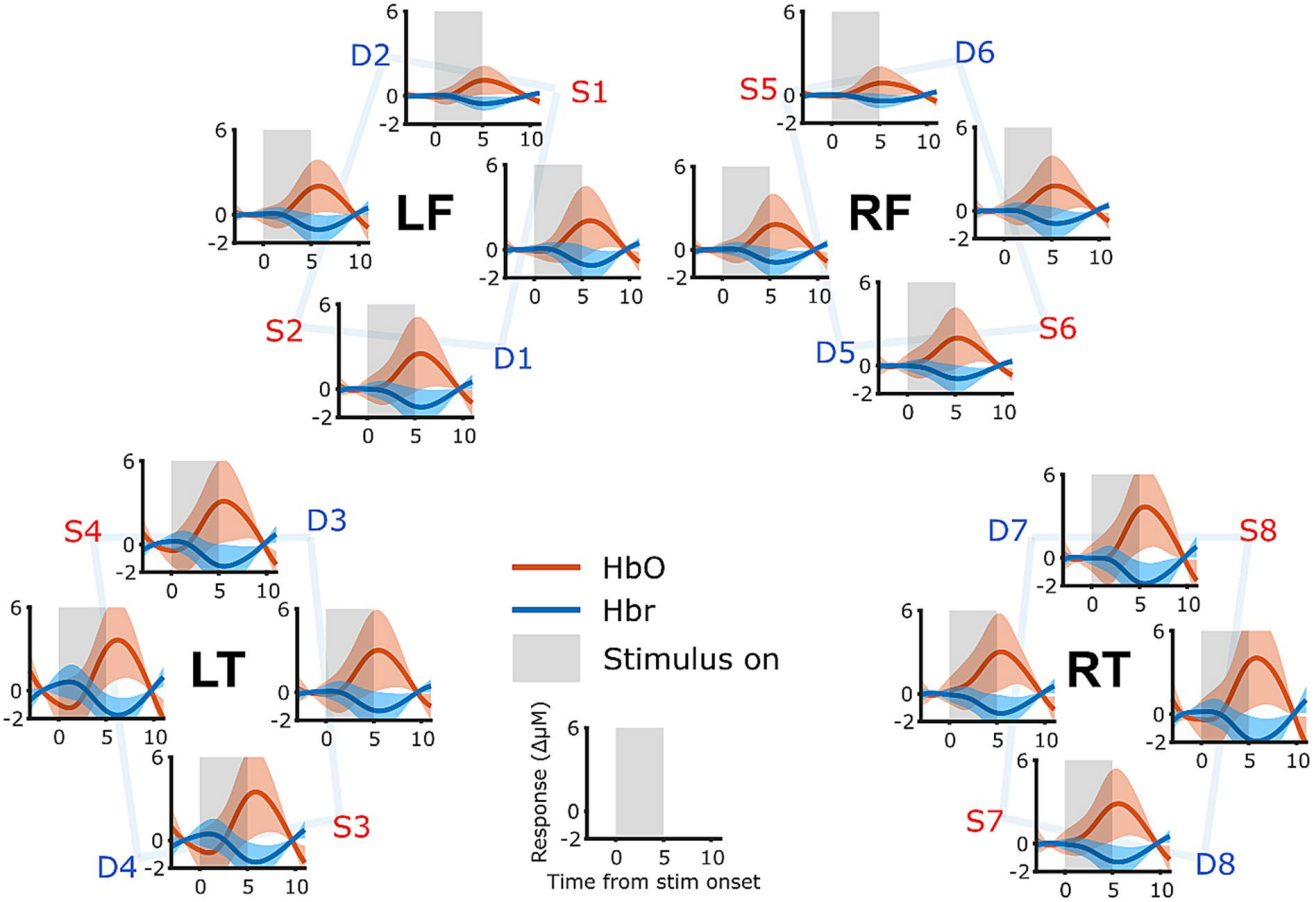
Grand average responses acquired at each recording channel. Each source and detector is indicated by red and blue text (S1–S8, D1–D8) respectively. Grey bars show when the sound stimulus was presented (five seconds duration), and shaded bars show one standard deviation across participants. Each region of interest is indicated in black text (LF, LT, RF and RT for left prefrontal, left temporal, right prefrontal, right temporal respectively) and consists of groups of four channels in their corresponding locations.

To estimate the size of the response in each epoch, a weighted average of the response was calculated. The weighting window was a Gaussian function centered on 5.5 s after stimulus onset with a standard deviation of 1.5 s and was based on the grand average responses shown in Fig. 5. This weighting function was multiplied with the HbO data and then averaged to give a single value (referred to hereafter as response size) for each epoch. These response sizes were used in all subsequent statistical analyses. The response size yields a similar value to the more familiar average taken within a specified time window and is used here because of its increased specificity to the expected response shape.

Synthetic no-response (control) epochs were generated for each participant by jittering the triggers marking the stimulus onset times by a random value uniformly distributed between − 10 and + 10 s before extracting epochs. Thus, the same number of synthetic no-response epochs were generated as there were real response epochs for each participant. These synthetic controls were used to estimate false positive rates (claiming a statistically significant response when none was present) in later analyses. Since the synthetic data contain more variance than real no-response data due to potential presence of actual responses (albeit not time-locked), the estimated false positive rates are likely to represent worst-case scenarios. We chose to use synthetic controls as inserting experimental control (no-stimulus conditions) would lengthen the experiment significantly, decreasing the amount of response data we could acquire before the baby awoke.

Statistical analyses were selected appropriately based on the hypothesis to be tested. We chose a linear-mixed model for group level analyses concerning response size changes due to different presentation conditions and regions of interest, as well as changes due to different speech token pairs. For individual level analyses, we chose non-parametric analyses to avoid any confounds due to non-normality of the data.

## Results

The fNIRS data were first explored by averaging epochs across participants, conditions and runs for each channel, giving an overview of the response morphology to speech sounds at each source-detector pair location (Fig. 5). fNIRS responses were observable in all regions: right and left hemispheres, and prefrontal and temporal regions. As the responses were of similar morphology across channels, we performed a time-average across channels in each of the four regions of interest. We calculated that the grand average fNIRS response (across all channels and conditions) peaked at 5.58 s.

### Group level discrimination and habituation is represented by fNIRS responses

**Figure 6:**
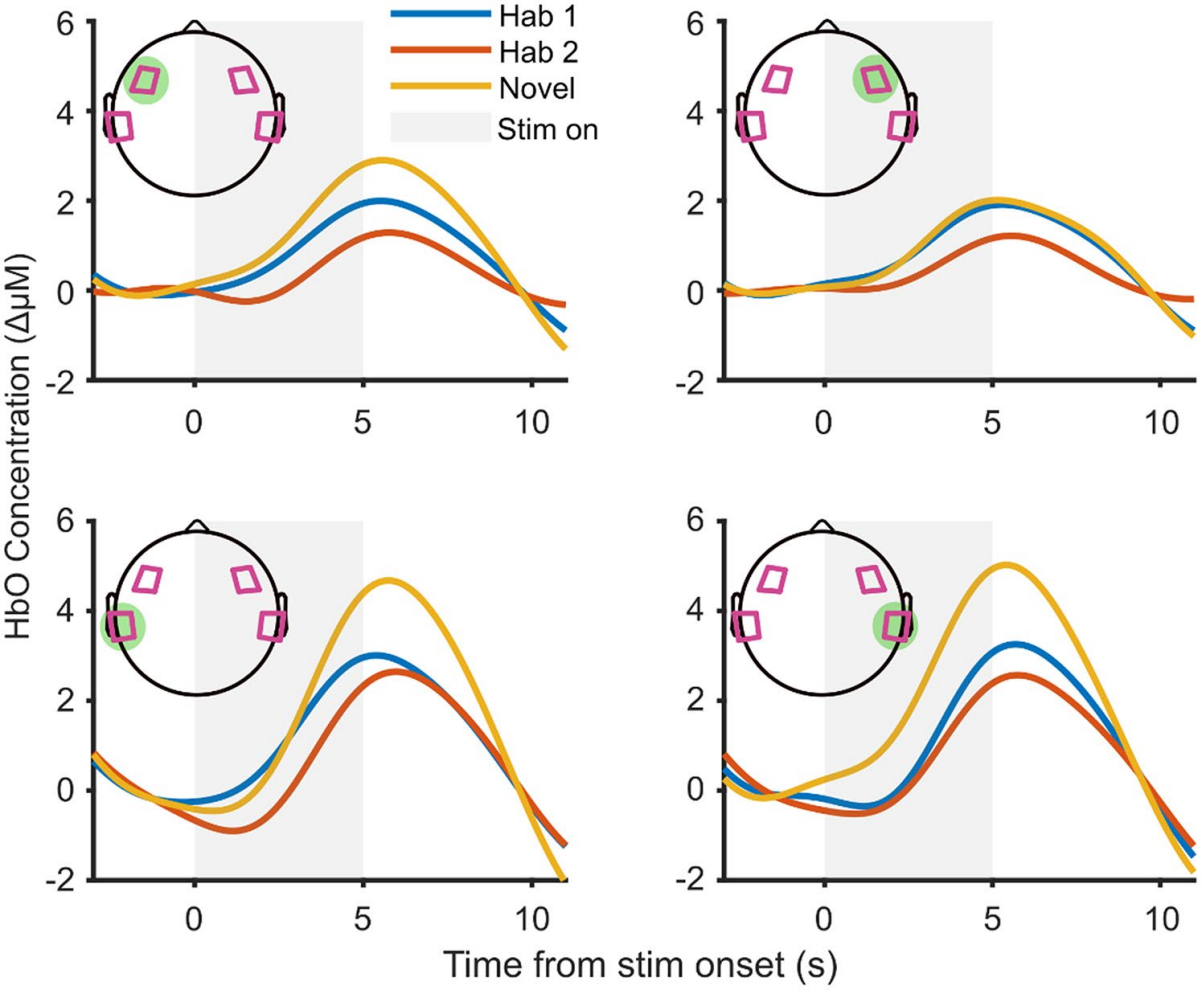
Group HbO responses at each ROI and to the Hab 1, Hab 2 and Novel stimulus conditions. Each ROI’s responses are in a different panel, with the ROI indicated by highlight on the head montage illustration. Each trace represents the response averaged across participants.

We tested two hypotheses on the group level responses at each brain region of interest: first, that the fNIRS response in the “Novel” condition was greater than that in the “Hab 2” condition, demonstrating significant discrimination between the two speech tokens; second, that the fNIRS responses in the “Hab 1” condition was greater than that in the “Hab 2” condition, demonstrating habituation to repeats of the same speech token. To do so, we used the participant-averaged responses as shown in Fig. 6. These responses were generated by first averaging across repetitions per participant, then across all participants, thus generating a trace for each condition (Hab 1, Hab 2 and Novel) and each region of interest.

To test our hypotheses, we fit a linear mixed model (Minitab 18.1) to the response sizes, with participants as a random factor, and regions of interest and condition and their interaction as fixed factors. The model revealed a significant effect of condition (*F*_2,242_ = 23.34, *p* < 0.0005) and of region of interest (*F*_3,242_ = 16.69, *p* < 0.0005) on the response sizes, but no significant interaction between condition and region of interest (*F*_6,242_ = 1.45, *p* = 0.197) (i.e., no region is better than another at differentiating responses to different conditions).

Post hoc Tukey Test (Minitab 18.1) showed that the three conditions were all significantly different, with “Novel” condition having the largest response (mean = 4.28), followed by the “Hab 1” condition (mean = 2.93) and then the “Hab 2” condition (mean = 2.09). Responses to “Novel” were significantly larger than to “Hab 2”, supporting the first hypothesis that discrimination is measurable on a group level in infants using fNIRS. Responses to “Hab 2” were significantly smaller than “Hab 1”, supporting the second hypothesis that fNIRS responses to repeated speech tokens habituate.

A post-hoc Tukey test showed that the temporal regions of interest gave significantly larger fNIRS responses (mean = 3.87, 4.16 for left and right temporal regions respectively) than the prefrontal regions (mean = 2.36, 1.99 for left and right prefrontal regions respectively), but there was no significant difference between the left and right hemispheres for either region.

Since there was no significant interaction between condition and region of interest, we averaged the responses across all regions of interest in each participant to increase statistical power when performing further analyses on the discrimination responses in individual infants.

### No significant effect of speech token contrast on discrimination response size

When the experiment was designed, we chose speech token contrast pairs that differed in the number and type of cues required for discrimination. The aim was to examine whether different contrast pairs would result in different magnitudes of the discrimination responses. We defined the “discrimination response size” as the difference between the responses to the “Novel” and “Hab 2” conditions.

**Figure 7:**
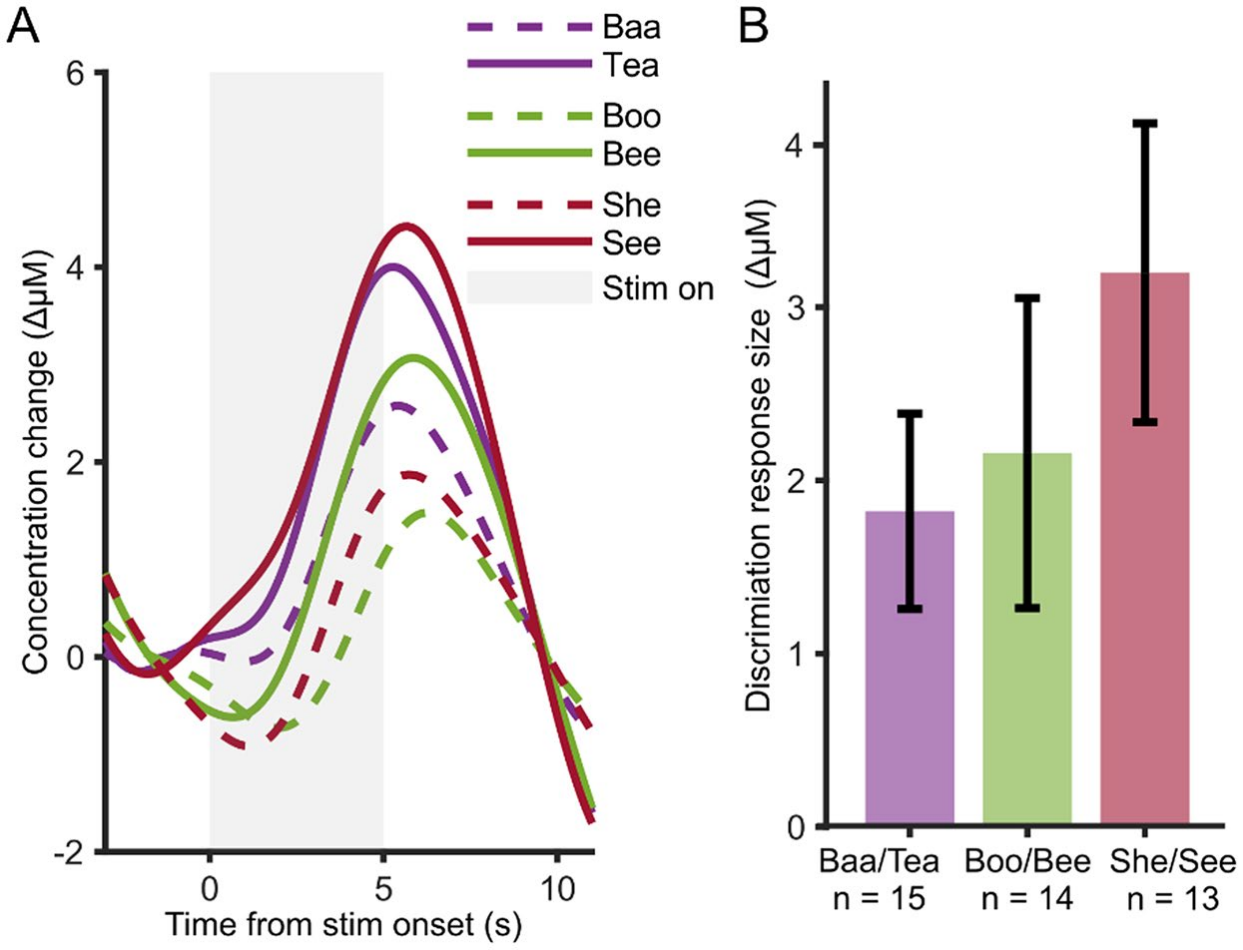
Difference between “Novel” and “Hab 2” conditions for each speech token contrast pair. (**A**) Shows the group average responses for each speech token (one color each); the “Hab 2”and “Novel" responses are represented by the broken and solid traces respectively. (**B**) Shows the calculated response size difference as a bar graph; error bars are one standard error of the mean across participants. Labels on x-axis indicates the number of participants who were delivered that particular speech token pair stimuli. There is no significant difference between discrimination response sizes.

Figure 7 shows the averaged responses (across participants and regions of interest) to the “Hab 2” and “Novel” conditions for each of the three speech token pairs. We fitted a mixed effects model on the discrimination response sizes, this time with participants as a random factor and speech token pair as a fixed factor. The model revealed no significant effect of the speech token pairs (*F*_2,39_ = 0.84, *p* = 0.44) for this group of normal-hearing infants. In fact, the non-significant trend was for the response to be larger for more difficult contrasts—opposite to our initial hypothesis.

### Characterizing performance of individual participant speech detection and discrimination assessment using fNIRS responses

One of the ultimate aims of our research is to develop a clinically relevant tool for hearing assessment using fNIRS. For the tool to be clinically relevant, speech detection and discrimination using fNIRS must be performed on an individual basis. Therefore, we used the fNIRS responses from each individual participant to measure whether their overall response to speech was present (speech detection) and whether their responses to “Novel” and “Hab 2” conditions were different (speech discrimination). We made the assumption that, as we were testing normally hearing infants, all were able to detect and discriminate speech tokens perceptually, and we used this assumption to evaluate the accuracy of our test outcomes. To evaluate the false positive rates, we used the simulated control responses generated as described in the “Materials and methods” section. Additionally, since the discrimination response size was not significantly different for different speech contrasts, we combined data for the speech token pairs each infant received for the discrimination analysis to increase statistical power.

**Figure 8:**
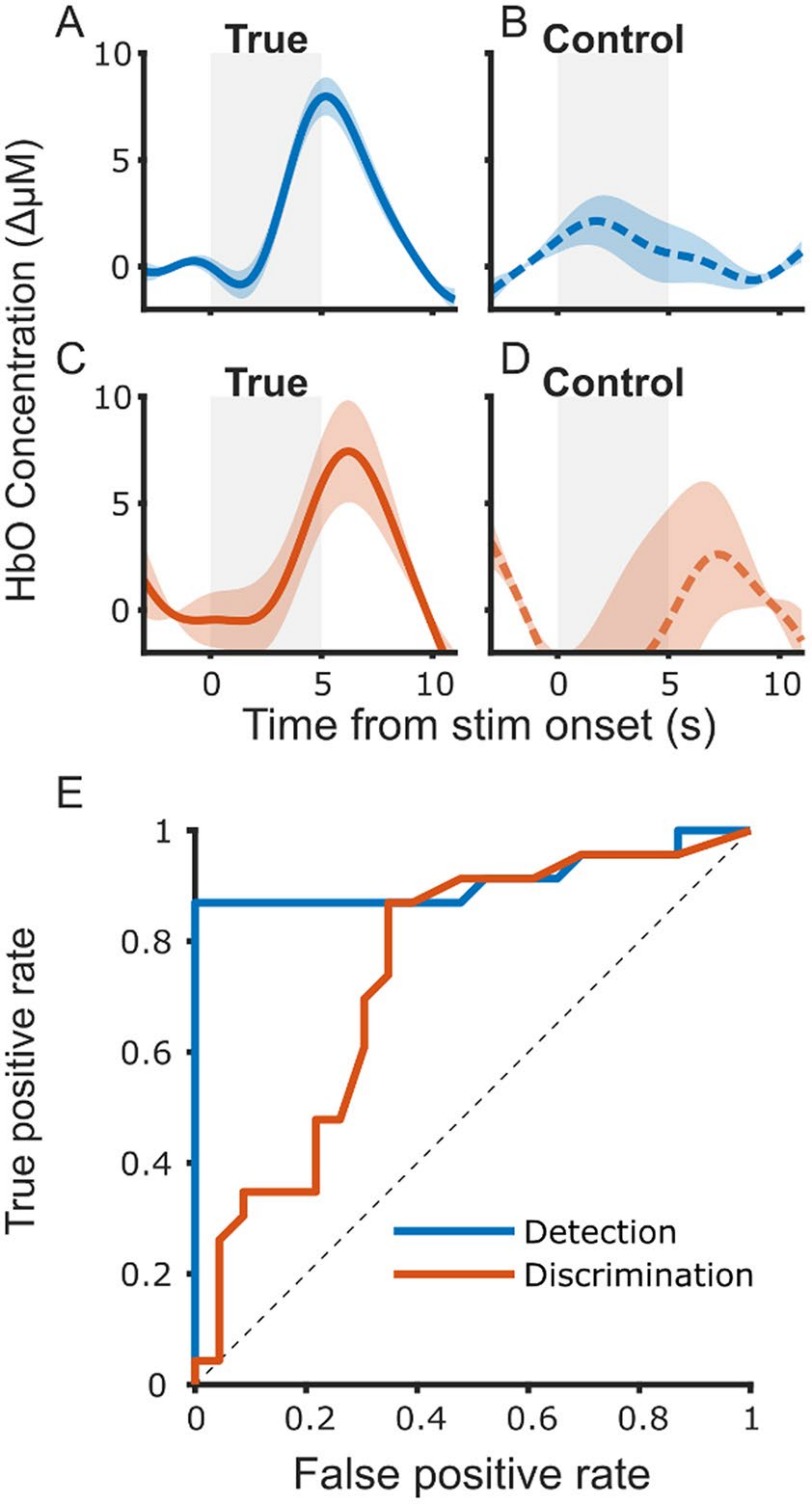
Individual infant detection and discrimination analysis. (**A,B**) Show examples from one participant SD011 of detection responses averaged across all channels and conditions for true (**A**) and control (**B**) stimulus blocks (shaded areas are 1 SEM). (**C,D**) Show the true and control discrimination responses respectively. (**E**) Shows the ROC curve, calculated across all 23 participants, for detection and discrimination response discovery respectively.

Figure 8 Panels A–D show an example of speech detection and discrimination assessment for one participant, SD011. We tested speech detection by combining all responses across conditions for this participant; panel A shows the averaged epoch across all responses. For each of these epochs, a response size was calculated (using the weighted average window), and a Wilcoxon signed rank test (*signrank*, Matlab 2020a) with a right sided tail (response is greater than zero) showed that the median response size was significantly greater than zero (*Z*_detection_ = 6.26, *p* < 0.0001) and indicated significant speech sound detection for this infant. We chose non-parametric tests for both detection and discrimination testing as there was no guarantee of normality in individual participants’ responses. For example, long-term adaptation would reduce the response sizes over the duration of the experiment (see next section).

To test whether SD011’s data would yield statistically significant detection responses with the simulated control (no response) blocks (i.e., false positive analysis), we performed the same analysis on the control data. The time-averaged epochs can be seen in Fig. 8 panel B. For SD011, the control analyses returned *Z*_detection_ =  − 0.41, *p* = 0.66, yielding a true negative result.

For the same infant (SD011), we tested speech discrimination by comparing the “Hab 2” and “Novel” conditions. Panel C shows the averaged difference epoch (response to “Novel” minus response to “Hab 2”). To test statistically for discrimination, a response size was calculated for each “Hab 2” and “Novel” stimulus and an unpaired Wilcoxon rank sum test (*ranksum,* Matlab 2020a) with a right sided tail was performed on the two groups response sizes to test whether responses to “Novel” had a larger median value than those to “Hab 2”. For this infant, the median response to “Novel” was significantly greater than the median response to “Hab 2” (*Z*_discrimination_ = 2.31, *p* = 0.0105) and thus indicated statistically significant speech sound discrimination. The control response analysis showed, *Z*_discrimination_ = 0.28, *p* = 0.39, indicating a true negative result for speech discrimination.

Finally, we performed a receiver operating characteristic (ROC) analysis by obtaining the individual response discovery result in each of the 23 participants, collating the true positive and false positive results, and then varying the critical *p* value for the discovery of a response. For example, when the critical *p* value is set to 0.05, 20 of 23 infants’ true detection responses were correctly identified (i.e., 87% true positive rate/sensitivity), while none of the 23’s control detection responses were identified (i.e., 0% false positive rate/100% specificity). Similarly, with critical *p* value of 0.05, 8 of the 23 infants’ true discrimination responses were correctly identified (35% true positive) and 2 of the 23 control discrimination responses were falsely identified as a real response (9% false positive). Figure 8 Panel E shows the ROC curve for detection and discrimination respectively, allowing for control of increased sensitivity by trading off a higher false positive rate. The ROC curve demonstrates a relatively better performance for detection over discrimination analysis, mainly in the low false-positives region. This is an indication that an improved speech discrimination algorithm and/or stimulus presentation protocol is required for clinical use.

### Response adaptation throughout experiment duration

During our data collection, we attempted variations of the run repetition sequence with an aim to maximize response sizes and thus improve the accuracy of observing fNIRS responses. In particular, we wanted to limit long-term adaptation: although short-term habituation in “Hab 2” *within* runs allowed discrimination responses to be detected, long-term adaptation *across* runs can potentially reduce the fNIRS response size, and signal-to-noise ratio, across the whole experiment. One strategy was to alternate different speech contrast pairs in each run to help maintain the novelty of the sounds at the start of each run. For infants SD007 to SD023 (*n* = 14), their experimental runs alternated between two speech token contrast pairs, whereas experimental runs for infants SD024 to SD029 (n = 6) used only one speech token contrast pair throughout the whole experiment.

**Figure 9:**
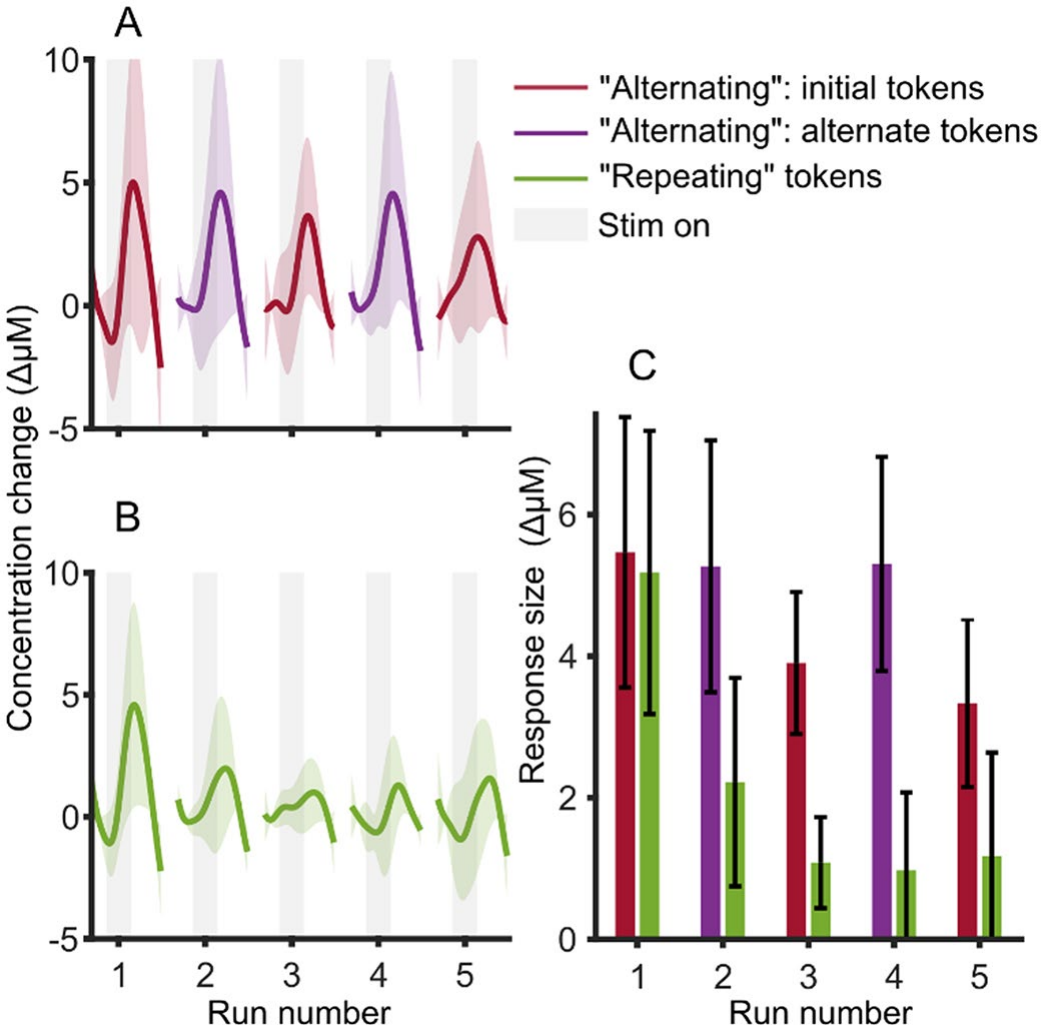
Comparison of responses to two sound presentation strategies. (**A**) Shows the “alternating” protocol, where two speech token pairs are presented in alternating runs. (**B**) Shows the “repeating” protocol, where the same speech token pair was repeated in five sequential runs. Shaded error bars show one standard deviation across participants. (**C**) Shows the calculated response sizes for both protocols as bar plots, with error bars showing one standard error of the mean; test statistics were calculated with these response sizes.

Each of these infants was presented with a minimum of five runs. Therefore, we chose to plot and analyze response sizes in the first five runs. “Post-Nov” data were excluded from the analysis since only one of the two groups had this condition in each run. Figure 9 shows the group average across all ROIs and also over “Hab 1”, “Hab 2” and “Novel” for all infants with either “alternating” or “repeating” runs. As infants were tested exclusively using either protocol, and there is a large between-participant variability, we chose to perform within-protocol comparisons: i.e., we tested whether the response size significantly decreased with repeated runs. To make the most equivalent comparison, we compared run one to runs three and five in the “alternating” protocol (i.e., the first, second and third repeats of the initial speech tokens) and compared run one to runs two and three in the “repeating” protocol (again, this is the first, second and third repeats of the same speech token).

We performed a *signrank* test (paired, non-parametric) where the alternate hypothesis is that the response size medians across participants are different between the runs that are being compared. Although Fig. 9 visually shows a pronounced reduction in average response size in the “repeating” protocol, the reduction in response size did not reach statistical significance for either protocol, although neared significance for the “repeating protocol”: for the “alternating” protocol, between run one and run three (*W* = 66, *p* = 0.213) or run one and run five (*W* = 67, *p* = 0.196); for the “repeating protocol”, between run one and run two (*W* = 18, *p* = 0.078) or run one and run three (*W* = 18, p = 0.078).

We repeated this analysis to see if the discrimination response size (difference between “Novel” an “Hab 2”) was reduced by the repeating of runs, and found no significant reduction in the alternating protocol of run one versus run three (*W* = 69, *p* = 0.163) or run five (*W* = 68, *p* = 0.179), nor in the repeating protocol of run one versus run two (*W* = 6, *p* = 0.844) or run three (*W* = 7, *p* = 0.781). Results for both adaption in detection and discrimination are shown in Table 1.

**Table 1:** Statistical testing output on whether there was significant reduction in response amplitude due to long-term adaptation.

	Detection habituation	Discrimination habituation
**Alternating protocol**		
Run 1 vs run 3	*W* = 66, *p* = 0.213	*W* = 69, *p* = 0.163
Run 1 vs run 5	*W* = 67, *p* = 0.196	*W* = 68, *p* = 0.179
**Repeating protocol**		
Run 1 vs run 2	*W* = 18, p = 0.078	*W* = 6, *p* = 0.844
Run 1 vs run 3	*W* = 18, p = 0.078	*W* = 7, *p* = 0.781

Comparisons between runs are made as illustrated in Fig. 9; A *signed-rank* test was run and the test statistic and *p* values are presented.

## Discussion

### Group results

At a group level, we showed that both temporal and prefrontal areas contained measurable fNIRS responses reflecting speech detection and discrimination in sleeping infants. Speech sound detection was reflected by a canonical response (increase in HbO and decrease in Hbr) across the entire temporal and prefrontal regions of the scalp, with the response being larger in the temporal region. This result is in keeping with other researchers who have also found a detection response over both regions^9,24,37,38^. Similarly, the discrimination response was present in both temporal and prefrontal regions, but with no statistically significant different in response size across regions or hemispheres. This finding is consistent with the results of other studies measuring speech discrimination using a combination of channels from temporal and prefrontal areas^10,39,40^, although others have suggested that prefrontal areas best reflect speech sound discrimination^9,41^.

Our group results replicate the findings of Nakano et al.^9^, while slightly adapting their stimulus presentation protocol to make the experimental run shorter and using multiple run repeats. While both studies showed significant detection and discrimination responses in both temporal and prefrontal regions, we also compared response size in different regions and found greater detection responses in the temporal compared to prefrontal regions, and a non-significant difference in the discrimination response size between temporal and prefrontal regions. Although Nakano et al.^9^ did not statistically compare the size of responses between regions of interest, their plot of grand average response size and standard errors in different regions and conditions (Fig. 8B in that paper) shows a large increase in response size in temporal compared to prefrontal regions that is relatively constant across stimulation conditions (Hab1, Hab2, Hab3, Test (novel), Post-Test), and a discrimination response size (Test minus Hab3 responses or change versus no-change in Test condition) that did not differ significantly across regions based on the standard errors provided: both observations consistent with our findings. Nakano et al.^9^ suggested that the discrimination response was preferentially located in the prefrontal region as they found only two individual channels (both prefrontal region) showed significant difference between change and no-change control responses in the test condition after correcting for multiple comparisons. However, this analysis does not test whether the discrimination response differs in temporal and prefrontal regions (a difference in significance does not imply a significant difference), and their group responses are entirely consistent with the ones found in our study, showing large discrimination responses in both regions.

It is interesting to note that the discrimination response was evident in these normally-hearing infants, even for the most subtle acoustic cue of “She” versus “See” that we used. Consistently, the Nakano et al.^9^ study showed robust group discrimination responses to the discrimination of “pa” versus “ba”, in which the only cue is the voicing onset time of the syllable. The finding that the most subtle acoustic contrast (She/See) did not evoke a smaller discrimination response in this study than the other contrasts suggests that an fNIRS discrimination test could be quite sensitive to even very subtle acoustic cues and thus be of broad utility for both research applications and potentially for personalized clinical management of hearing-impaired infants. In our group of normally-hearing infants, we could not reject the null hypothesis that the discrimination response was equal for different speech contrasts. This result could be due to lack of power due to large variance across infants, or it could be that all the contrasts we used were relatively easy for this participant group to discriminate. It remains to be seen, however, whether the discrimination response in hearing-impaired infants differs in size or characteristics from that in normally-hearing infants.

In this study, we presented numerous runs for each baby so that statistical tests could be carried out for individual infants. In general, adding more data to an average can improve the signal-to-noise ratio and consequent detection efficiency. However, if long-term adaptation occurs over a lengthy test session, the size of the response will decrease, potentially having an opposite effect on the signal-to-noise ratio. Although adaptation across multiple runs failed to reach statistical significance in our data, there was a very strong trend (p = 0.078) of response reduction in the smaller group of 6 infants who were presented with the same speech contrast in every run. However, due to the small number of infants, the test lacked statistical power. This trend was not evident in the larger group that received alternating speech contrast pairs in each run, making it likely that this strategy helped to maintain the response amplitudes over the test session. However, in a practical sense, this strategy is only clinically useful if testing more than one contrast pair, and it may require double the testing time to obtain sufficient epochs for each of the contrast pairs.

While care was taken to ensure the cap placement is consistent in every participant, due to the fast brain development in infants, and the relatively wide age range (2 to 10 months) in this study, there may be variability in the data recorded in terms of channel position and the underlying imaged brain region. The main effect of the variability would be the impact on the weighted average window used to calculate response sizes. We believe that the variation would not have a great impact in terms of the group level findings since the infant responses are similar across brain regions^24^; it is possible that individualized response shapes can be generated on a per-participant basis to improve individual-level results as future work.

### Testing for detection and discrimination in individual infants

In this study, we explored whether an adaptation of the habituation/dishabituation method of Nakano et al.^9^ could yield reliable detection and discrimination results in individual infants. At an individual level (with α = 0.05), we found statistically significant speech sound detection responses in 87% of babies and significant discrimination responses in 35% of babies, with the control non-stimuli yielding false discovery rates (0% and 9% respectively) consistent with the theoretically expected 5%. As a first pass, and using very simple statistics, these results show great potential for clinical utility, but that, for discrimination especially, improvements are needed to achieve accuracy sufficient for guiding clinical management in individuals with a hearing impairment.

There are two alternative types of modification that could potentially improve the accuracy of the fNIRS test of speech discrimination: developing an improved test protocol; and utilizing more sophisticated statistical techniques. The test protocol we used in this study has several disadvantages for its use in repeated test runs. Behavioral studies of speech discrimination in infants that rely on the habituation/dishabituation technique have shown the critical importance of maintaining the novelty of the novel stimulus if multiple presentations are needed when testing an individual child. For example, Houston et al.^42^ showed that increasing both the complexity of the novel stimulus, and increasing its oddity (or rarity), together significantly increased the changes of individually-significant discrimination being found. In the procedure we used here, the novel sound occurred in one quarter or one third of all stimulus blocks, and in groups of five blocks, and so potentially lost novelty as the test progressed. It is therefore possible that similar strategies as those used by Houston et al.^42^ could be applied to the fNIRS case to greatly improve the response to the novel speech sound.

The statistical analysis that we applied to the individual infants in this study was a non-parametric test on the response sizes. While this is a conservative approach that does not rely on an assumption that the true response sizes are consistent across epochs, a more powerful approach to improve signal detection might be achieved by building a model of the expected response distribution over time in infants, although this approach may only be realistic with data from many more infants than took part in this study. A model can make use of some distinctive characteristics of the responses. For example, short-term habituation and long-term adaption of the responses means we can expect true responses to vary in amplitude over time in a predictable manner, both within-test runs and between-test runs. These expected characteristics of the responses would allow additional constraints to be placed on a model and could potentially be exploited by a novel algorithm to increase accuracy or reduce testing time. Our group is currently working on these two types of optimizations, with the aim of achieving accuracy and reliability in individual infants (with and without hearing impairment) that would support the use of fNIRS in pediatric audiology clinics to guide clinical management decisions.

### Limitations

In this study, we had insufficient statistical power to compare the efficacy different stimulus protocols in detail. Instead, our main questions revolved around how repeating test runs of the same protocol would influence the ability to test detection and discrimination in individual infants. If difficulty in recruitment was not a limitation, and given a larger cohort of infant participants, it is possible that we might observe additional significant effects that were only trends in our current data. Namely, it is possible that there is an interaction effect between brain regions of interest and the stimulation condition (Fig. 6, right frontal region’s responses are quite similar); there may also be a difference discrimination response for different speech token contrasts (Fig. 7, “She/See” pairing has discrimination response); finally, differences in long-term adaptation due to the different protocol may also become more pronounced. With larger test numbers, it would also be interesting to examine age-related changes (using either a cross-sectional or longitudinal study design), as it is well known that EEG-measured cortical responses in infants mature in morphology over time^43^. Any age differences in fNIRS responses may be a confound to be aware of, or a useful feature, in studies such as this one.

## Conclusions

In this study, we have presented a test procedure that aimed to produce an objective measure of infant speech detection and discrimination using fNIRS responses for individual sleeping infants. Robust detection and discrimination results were obtained at a group level. When the test procedure and analysis was performed on individual infants with no hearing impairment, we could measure fNIRS responses to speech detection in 87% of individual infants, and to speech discrimination in 35% of individual infants. The results show a promising first step towards a novel, objective audiological test of speech detection and detection ability which will allow earlier intervention for infants who have hearing impairment. Current work is aiming to improve reliability and accuracy above the reported levels by use of a novel sound presentation protocol and optimized fNIRS signal processing and analysis methods.

## Data Availability

The datasets generated during and/or analysed during the current study are available from t he corresponding author on reasonable request.
